# Phenolic acids and a static magnetic field change the expression of transforming growth factor β isoforms in amelanotic melanoma cells

**DOI:** 10.1007/s11033-023-08336-1

**Published:** 2023-03-10

**Authors:** Magdalena Kimsa-Dudek, Agnieszka Synowiec-Wojtarowicz, Agata Krawczyk

**Affiliations:** Department of Nutrigenomics and Bromatology, Faculty of Pharmaceutical Sciences in Sosnowiec, Medical University of Silesia, Katowice, Jednosci 8, Sosnowiec, 41-200 Poland

**Keywords:** Static magnetic field, cancer, Chlorogenic acid, Caffeic acid, Melanoma cells, Transforming growth factor β

## Abstract

**Background:**

Melanoma is an aggressive type of cancer that can metastasize to numerous other organs. TGFβ is one of the key signaling pathways in melanoma progression. Previous studies on various types of cancer have shown that both: polyphenols and a static magnetic field (SMF) can be potential chemopreventive/therapeutic agents. Therefore, the aim of the study was to evaluate the effect of a SMF and selected polyphenols on the transcriptional activity of TGFβ genes in melanoma cells.

**Methods and results:**

Experiments were performed on the C32 cell line treated with caffeic or chlorogenic acids, and with simultaneous exposure to a moderate-strength SMF. The RT-qPCR method was used to determine the mRNA level of genes encoding the TGFβ isoforms and their receptors. The concentration of the TGFβ1 and TGFβ2 proteins were also measured in the cell culture supernates. The first response of C32 melanoma cells to both factors is the reduction of TGFβ levels. Then, mRNA level of these molecules returned to values close to pre-treatment level by the end of experiment.

**Conclusion:**

Our study results demonstrate the potential of polyphenols and a moderate-strength SMF to support cancer therapy by altering TGFβ expression, which is a very promising topic for the diagnosis and treatment of melanoma.

## Introduction

Although cutaneous melanoma (CM) constitute a small proportion of skin cancers, it is the most common cause of cancer death in the young population [1]. According to studies, the incidence of this cancer has increased sharply among people in developed countries [2, 3]. It is a fairly aggressive type of cancer that, despite having the tumor removed, can metastasize to numerous other organs [4]. The etiopathogenesis of the disease is quite complex, however, it has not been fully documented yet. Changes at the molecular level are quite important [5, 6]. Multiple signaling pathways are involved in the development of CM. Mention is made of pathways: Jak/STAT (Janus kinase/signal transducer and activator of transcription), Wnt, NF-κB (nuclear factor kappa B), and also TGFβ (transforming growth factor β) [3, 7, 8]. Impact character of TGFβ is complex, depending on the type of tissue and the stage of the disease [9]. Although TGFβ is a strong inhibitor of epithelial cell proliferation, which is related to the inhibitory effect on the cell cycle [2], the final mechanism of action is quite controversial. It is mainly indicated that TGFβ is an ideal microenvironment for tumor development, promotes angiogenesis and the development of the immunosuppressive mechanism. This confirms previous reports on the secretion of TGFβ by most tumors, including melanomas [10]. It should also be taken into account that the mRNA level of individual TGFβ isoforms depends on the type and nature of cells. As reported in the literature, the mRNA transcript for TGFβ-2 is characteristic only for melanoma cells, while the transcripts for TGFβ-1 and TGFβ-3 - for normal and neoplastic cells. The protein products of TGFβ-2 and TGFβ-3 are expressed only by neoplastic cells and participate in melanoma progression [10].

In recent years, a lot of attention has been paid to substances that can help in the fight against this cancer. Many of them are already known, but the effects of substances of natural origin, commonly found in food products, are still being researched. There are more and more studies proving that more and more compounds of plant origin may constitute a new and important agent with pharmacological properties [11]. The latest literature data indicate a group of those that may show anti-inflammatory properties in terms of preventing the development of neoplastic diseases [12]. Such compounds include chlorogenic acid and caffeic acid.

The first is a derivative of a cinnamic acid, a compound structurally composed of caffeic acid and quinic acid. It is a polyphenol that is commonly found in the diet: apples, carrots, artichokes, etc. [13, 14]. It shows mainly anti-inflammatory, antioxidant, anti-neurodegenerative, but also anti-diabetic and anti-lipidemic effects [13, 15, 16]. However, the anti-cancer effect has been widely reported recently [17, 18]. It has been shown that this compound suppresses the proliferation of melanoma cells and reduces reactive oxygen species (ROS), which may reduce photocarcinogenesis [15].

The second compound is caffeic acid, i.e. 3,4-hydroccinamonic acid. It is found in many plants, including apples and coffee. It has antioxidant, antibacterial and anticancer properties [19]. Numerous studies show that caffeic acid inhibits the proliferation of neoplastic cells. Moreover, it has been shown that this compound has the potential to reduce metastases in the neoplastic process [20]. It should be noted that the exact mechanism of these properties has not been fully explained.

Melanocytes present in the skin, but also in the hair and eyes, protect against reactive oxygen species and UV radiation. Consequently, chlorogenic acid and caffeic acid are commonly used in cosmetic products [21].

In recent years, additional attention has been paid to other factors that may support the action of active substances. Among them, there is a static magnetic field (SMF), which is being borne in mind by numerous scientific studies [22, 23]. Research is underway to draw attention to its potential use in medicine. The action of this factor is discussed in terms of its impact on cellular processes important in the pathogenesis of a given disease entity. It should be noted that in some types of cancer it was possible to confirm its potential therapeutic importance in vitro [24] and in vivo [25]. Moreover, there are indications that it may act synergistically with chemotherapeutic agents [25]. Currently, there are no described studies on the direct effect of a SMF with natural substances on various cancer cells, while those focusing on the use of magnetic nanoparticles activated by a magnetic field (MNP) in the treatment of cancer seem to be promising [26, 27]. Therefore, it seems important to analyse the effect of a SMF in the aspect of the application of a bioactive substance derived from food in order to demonstrate their potential anticancer activity.

## Materials and methods

Experiments were performed on the C32 cell line (amelanotic melanoma, ATCC, CRL-1585; Manassas, VA, USA) treated with caffeic (CA) or chlorogenic (CGA) acids, and with simultaneous exposure to a moderate-strength static magnetic field.

### Cell culture conditions

The cell cultures were performed in Dulbecco’s Modified Eagle Medium (DMEM, Lonza, Basel, Switzerland) enriched with 10% FBS (fetal bovine serum, Sigma-Aldrich, St. Louis, MO, USA), amphotericin B (0.25 mg/ml), and penicillin (10,000 U/ml) in an incubator (Heraeus, Hanau, Germany) with the atmosphere of 5% CO^2^ at 37 °C. The Countess™ Automated Cell Counter (Invitrogen, Carlsbad, CA, USA) was used to assess the number and viability of cells. Cells between passages four and eight and at approximately 80% confluency for these experiments were used.

The experiment was conducted under the conditions described previously [28]. The C32 cells were seeded in 25 cm^2^ cell culture flasks (Sarstedt, Nümbrecht, Germany) and after 24 h of incubation, the cells were treated with chlorogenic acid (CGA, Sigma-Aldrich, St. Louis, MO, USA) or caffeic acid (CA, Sigma-Aldrich, St. Louis, MO, USA) at a concentration of 1 mmol/L with simultaneous exposure to a moderate-intensity static magnetic field (0.7 T) and cultured in an incubator with a humidified atmosphere of 95% air and 5% CO_2_ at 37 °C. Control cells and cells that had been treated with phenolic acids and/or SMF were cultured simultaneously under the same conditions. Following next 24 h of incubation, cells were washed in PBS (Phosphate Buffered Saline, Lonza, Basel, Switzerland) and pelleted to further molecular analysis. At the same time, the culture medium was also harvested. Samples were stored at -80 °C until further analysis. Separate exposures (in triplicate) for analyses were performed.

**Fig. 1:**
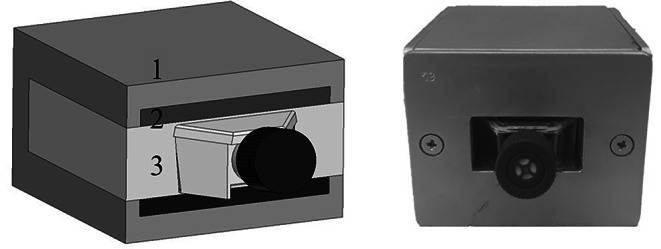
Magnetic test chamber for in vitro culture of cells exposed to a static magnetic field (1 – ferromagnetic yoke; 2 – permanent magnets; 3 – non-magnetic material)

In our experiments, a static magnetic field was generated in a special magnetic chambers consisting of a ferromagnetic yoke and permanent magnet, and fully fitted to the culture flasks (Fig. 1). In control chambers that were used to culture the unexposed cells, the steel (0.0 T) was used instead of permanent magnet [29, 30]. The design of these magnetic chambers allow for uniform distribution of magnetic flux density over the measurement space of the flask. The flux densities in the chambers were measured using a gauss meter.

The intensity of field was selected on the based on the previous results that showed the possibility of its use in a cancer treatment [28, 31]. Also the concentration of the phenolic acids was selected on the based on the previous research that showed their cytotoxic properties and the ability to induce apoptosis of melanoma cells while normal cells were not affected [28, 32, 33].

### RNA extraction

The RNA extraction was performed using a TRIzol reagent (Invitrogen, Carlsbad, CA, USA) that is specifically formulated to isolate total RNA from many sample types. The procedure was performed according to the manufacturer’s instructions and consisted of the following stages: cell lysis, phase separation, RNA precipitation, RNA wash and RNA dissolving. In the next step, agarose gel electrophoresis and MaestroNano MN-913 (MaestroGen Inc., Las Vegas, NV, USA) spectrophotometer was used for the qualitative and quantitative assessment of RNA extracts.

### Real time RT-qPCR assay

The RT-qPCR method (reverse transcription-quantitative polymerase chain reaction) was used to determine the mRNA level of genes encoding the *TGFβ* isoforms (*TGFβ-1, TGFβ-2* and *TGFβ-3*) and their receptors (*TGFβ-R1, TGFβ-R2* and *TGFβ-R3*). The gene expression were evaluated with SYBR Green I chemistry (SensiFAST SYBR No-ROX One-Step, Bioline, London, UK) and a LightCycler® 480 Instrument II (Roche Life Science, Basel, Switzerland). The oligonucleotide specific primers were commercially available (Sigma-Aldrich, St. Louis, MO, USA). Each reaction was repeated three times. The thermal conditions of the reaction were as follows: reverse transcription, 45 °C − 10 min; activation, 95 °C − 2 min; 45 cycles: denaturation, 95 °C − 5 s; annealing, 60 °C − 10 s and extension, 72 °C − 5 s. At the end melting curve analysis was also performed.

2^−(ΔCt)^ method with β-actin as a reference gene (where ΔCt = Ct of our gene of interest - Ct of β-actin) was used to determine the relative gene expression [34].

### ELISA assay

The concentration of the TGFβ1 and TGFβ2 proteins were measured in the acid-activated cell culture supernates using the Quantikine Human TGFβ1 Immunoassay and the Quantikine Human TGFβ2 Immunoassay kits (R&D Systems, Minneapolis, MN, USA) according to the manufacturer’s protocol. The absorbance at a wavelength of 450 nm was read on a Wallac 1420 VICTOR microplate reader (PerkinElmer, Waltham, MA, USA). Simultaneously, the TGFβ concentration was measured in the control medium in order to assess the background level of TGFβ. All assays were performed in duplicate.

### Statistical analyses

The statistical analyses were performed using Statistica 13.3 software (StatSoft, Tulsa, OK, USA) and the level of significance was set at p < 0.05. In the statistical analyses, the one-way ANOVA and Tukey post hoc tests were used due to the normal distribution of the data.

## Results

In our experiment the changes in *TGFβ* gene expression and TGFβ protein concentration in C32 melanoma cells treated with caffeic or chlorogenic acid and simultaneously exposed to a static magnetic field were determined.

### Expression of ***TGFβ*** genes and their receptors in C32 melanoma cells

**Fig. 2:**
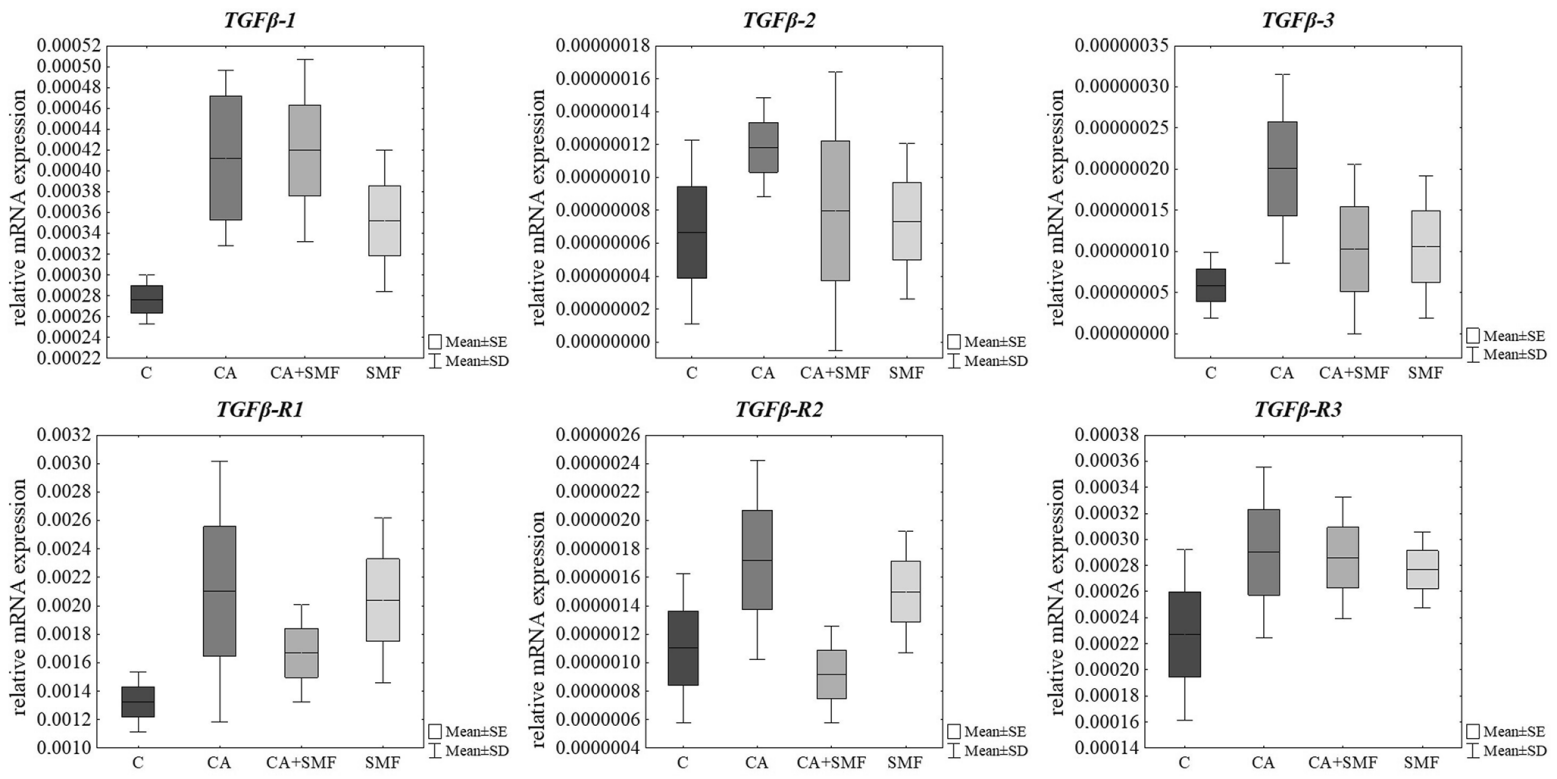
Relative mRNA expression of *TGFβ* isoforms and their receptors in cells that have been treated with caffeic acid and cells that have been exposed to a static magnetic field. The results are presented as a mean ± SD; C – control cells; CA – cells that have been treated with caffeic acid; SMF – cells that have been exposed to a static magnetic field; CA + SMF – cells that have simultaneously been exposed to caffeic acid and a static magnetic field

**Fig. 3:**
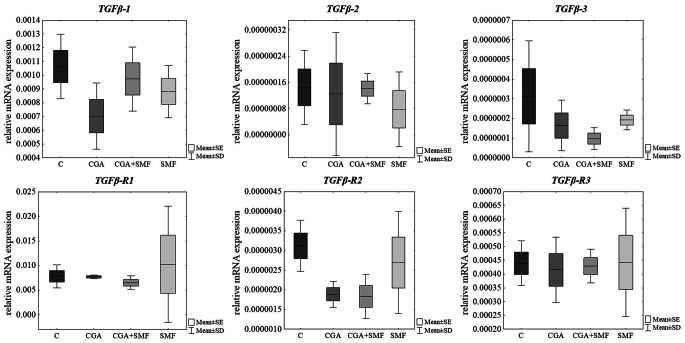
Relative mRNA expression of *TGFβ* isoforms and their receptors in cells that have been treated with chlorogenic acid and cells that have been exposed to a static magnetic field. The results are presented as a mean ± SD; C – control cells; CGA – cells that have been treated with chlorogenic acid; SMF – cells that have been exposed to a static magnetic field; CGA + SMF – cells that have simultaneously been exposed to chlorogenic acid and a static magnetic field

After 24 h of treatment of melanoma cells with a caffeic acid, with a 0.7 T-intensity static magnetic field and both factors no statistically significant alterations in expression of *TGFβ* isoforms and their receptors in comparison to control cells were observed (Table 1; Fig. 2). Similar results were found after treatment of C32 cells with chlorogenic acid and with simultaneous exposure to a static magnetic field (Table 1; Fig. 3).

**Table 1:** Relative mRNA expression of *TGFβ* isoforms and their receptors in C32 melanoma cells that have been treated with caffeic or chlorogenic acids and that have been exposed to a static magnetic field

Groups	*TGFβ-1*			*TGFβ-2*			*TGFβ-3*		
	FC	Expression	*p	FC	Expression	*p	FC	Expression	*p
**Caffeic acid**									
CA	1.49	↑	NS	1.77	↑	NS	3.41	↑	NS
CA + SMF	1.52	↑		1.19	↑		1.75	↑	
SMF	1.27	↑		1.10	↑		1.80	↑	
**Chlorogenic acid**									
CGA	-1.51	↓	NS	-1.16	↓	NS	-1.90	↓	NS
CGA + SMF	-1.09	↓		-1.03	↓		-3.22	↓	
SMF	-1.20	↓		-1.85	↓		-1.63	↓	
	***TGFβ-R1***			***TGFβ-R2***			***TGFβ-R3***		
	**FC**	**Expression**	*p	**FC**	**Expression**	*p	**FC**	**Expression**	*p
**Caffeic acid**									
CA	1.58	↑	NS	1.56	↑	NS	1.28	↑	NS
CA + SMF	1.26	↑		-1.20	↓		1.26	↑	
SMF	1.54	↑		1.36	↑		1.22	↑	
**Chlorogenic acid**									
CGA	-1.01	↓	NS	-1.65	↓	NS	-1.06	↓	NS
CGA + SMF	-1.20	↓		-1.70	↓		-1.02	↓	
SMF	1.31	↑		-1.16	↓		1.01	↑	

FC – fold change; ↑ - increased expression vs. control; ↓ - decreased expression vs. control;

NS- not significant, one-way ANOVA test;

CA or CGA – cells that have been treated with caffeic or chlorogenic acid;

SMF – cells that have been exposed to a static magnetic field;

CA or CGA + SMF – cells that have simultaneously been exposed to caffeic or chlorogenic acid and a static magnetic field

### Concentration of TGFβ proteins in C32 cell supernates

**Fig. 4:**
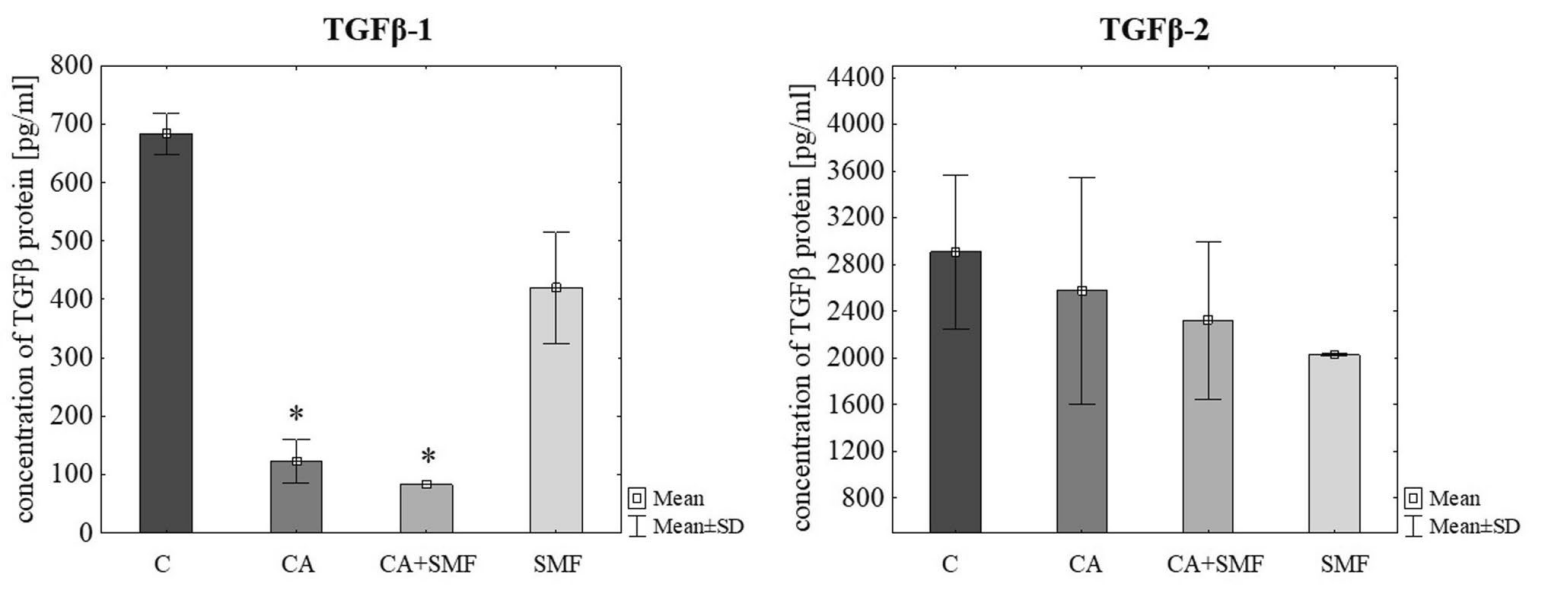
The TGFβ-1 and TGFβ-1 protein concentration in the C32 cells after exposure to the caffeic acid and a static magnetic field. The results are presented as a mean ± SD; C – control cells; CA – cells that have been treated with caffeic acid; SMF – cells that have been exposed to a static magnetic field; CA + SMF – cells that have simultaneously been exposed to caffeic acid and a static magnetic field; *p < 0.05 vs. C

At protein level it was found that caffeic acid and caffeic acid with simultaneous exposure to a moderate-strength static magnetic field cause a statistically significant reduction of TGFβ-1 molecule concentration in comparison to control cells (p = 0.009, p = 0.013, respectively, Tukey post hoc test). However, both factors did not have a significant effect on the concentration of TGFβ-2 protein (Table 2; Fig. 4).

**Table 2:** The TGFβ-1 and − 2 protein concentration in supernates of C32 melanoma cell cultures

Groups	TGFβ-1		TGFβ-2		
	FC	*p	FC	*p	
**Caffeic acid**					
CA	-5.57	0.008	-1.13	NS	
CA + SMF	-8.21		-1.25		
SMF	-1.63		-1.44		
**Chlorogenic acid**					
CGA	-1.34	0.013	-1.24	0.007	
CGA + SMF	-1.60		-1.35		
SMF	-3.45		-1.52		

FC – fold change;

*p < 0.05, NS- not significant, one-way ANOVA test

CA or CGA – cells that have been treated with caffeic or chlorogenic acid;

SMF – cells that have been exposed to a static magnetic field;

CA or CGA + SMF – cells that have simultaneously been exposed to caffeic or chlorogenic acid and a static magnetic field

**Fig. 5:**
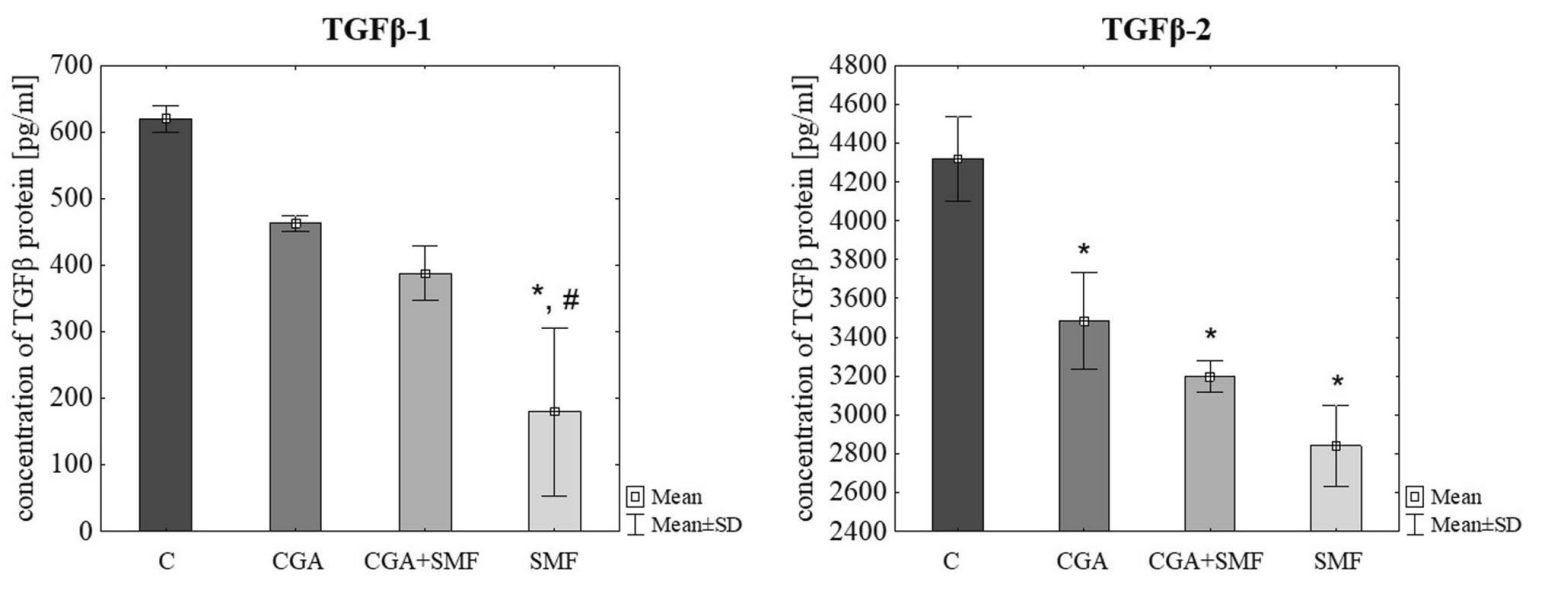
The TGFβ-1 and TGFβ-1 protein concentration in the C32 cells after exposure to the chlorogenic acid and a static magnetic field. The results are presented as a mean ± SD; C – control cells; CGA – cells that have been treated with chlorogenic acid; SMF – cells that have been exposed to a static magnetic field; CGA + SMF – cells that have simultaneously been exposed to chlorogenic acid and a static magnetic field; *p < 0.05 vs. C; #p < 0.05 vs. CGA

In case of chlorogenic acid there was indicated a significant reduction of TGFβ-1 protein concentration in cells that have been exposed to a SMF in comparison to control (p = 0.010, Tukey post hoc test). Statistically significant differences in the concentration of this protein were also observed between cells that have been treated with a SMF and cells that have been treated with a CGA (p = 0.045, Tukey post hoc test). There was also a statistically significant decrease in the concentration of TGFβ-2 molecule in CGA-treated cells, in SMF-exposed cells, and in cells that have been simultaneously treated with both factors (p = 0.045, p = 0.006, p = 0.017, respectively, Tukey post hoc test) (Table 2; Fig. 5).

Summarizing the obtained results, the first response of C32 melanoma cells to both factors (phenolic acids and a SMF) is the reduction of TGFβ levels, which is visible in 24 h of experiment at the protein level. Then, mRNA level of these molecules returned to values close to pre-treatment level by the end of experiment.

## Discussion

Considering the increasing number of patients diagnosed for melanoma and the still not fully known etiopathogenesis of this disease, newer and newer diagnostic markers of this cancer are being searched for. Our focus was the TGFβ pathway as a therapeutic target in the treatment of melanoma. Previous studies have shown the role of TGFβ in the development and progression of melanoma. Cesaro et al. [9] indicated the participation of TGFβ in tumor development. The research was carried out in terms of the role of the zinc finger protein ZNF224 in signaling the TGFβ pathway as a stimulator of the pro-oncogenic function of TGFβ favourable to epithelial-mesenchymal transition [9]. Moreover, Gipsyianti et al. [35] noted the participation of both TGFβ and cyclooxygenase- 2 (COX-2) in the development of acral melanoma (AM), as well as the depth and invasion of the lesion. The involvement of the TGFβ pathway in the development of melanoma has also been noted by Ren et al. [36]. In our research, we focused on the aspect of changes in TGFβ expression due to the use of plant compounds and the physical factor. The aim of our study was to find out whether these chemical and physical factors could potentially be used to support cancer therapy and limit the tumor development through the suppression of pro-tumorigenic TGFβ. Plant compounds are widely used in the treatment of various diseases, including cancer. In our previous research, we have already proved the positive effect of chlorogenic and caffeic acid and a static magnetic field on melanoma cells in the aspect of oxidative stress, cell redox homeostasis and apoptosis [28, 33]. In turn, Li et al. [37] demonstrated the effectiveness of treatment consisting of a combination of sialic acid modified chlorogenic acid liposome therapy (CA-SAL) in combination with anti-PD1 (anti-programmed death protein 1) antibody treatment. Moreover, the research by Choi et al. [38] also showed the positive effect of *Phyllostachys nigra* active compounds on melanoma cells. It is worth noting that the active ingredients of this plant include those we have studied: caffeic acid and chlorogenic acid. These acids are also the active ingredients of the tincture of European mistletoe (*Viscum album* L.). The authors showed that these compounds reduced the growth of tumor cells in a dose-dependent manner in murine melanoma cells [39].

In our study, we decided to check the effect of a SMF in combination with plant compounds on the reduction of tumor development as a result of modification of *TGFβ* expression. The topic is quite innovative due to the small amount of research on a SMF. It would seem that a SMF is applicable because of its documented performance. A SMF has been shown to influence tumor blood flow by reducing it and thus impair angiogenesis and growth of solid tumors in vivo. There are studies showing that exposure of mice treated with cis-diaminodichloroplatinum (cis-platinum) and (2-chloroethyl) tetrahydro-2 H-1,3,2-oxazaphosphite-2-N, N-bis 2-oxide -amines (cyclophosphamide) to a SMF contributed to the desired therapeutic effect [40]. But molecular mechanism its action on cells is not fully explained. On the other hand, there are recent reports showing that cell response cells to a magnetic field can be related to the radical pair mechanism [41, 42] and ion cyclotron resonance [43].

Polyphenols, including phenolic acids, as bioactive substances, can influence the expression of many genes and thus may regulate a number of signaling pathways in cells, including those related to TGFβ [44, 45]. Also, a SMF can affect signaling pathways by modifying the concentration of secondary messengers such as calcium ions, reactive oxygen species, and changing the biophysical properties of cell membranes [46]. In the study by Bekhite et al. [47], the researchers showed that a static magnetic field enhanced cardiomyocyte differentiation of mouse embryonic stem cells. The proposed mechanism of a SMF action is related with Ca^2+^ influx that is involved in reactive oxygen species production and induction of cardiomyogenesis. In turn, Novikov et al. [48] observed that combined magnetic field (CMF) caused a decrease of the respiratory burst in neutrophils probably through the effect of a CMF at the resonance frequency of Fe^3+^. Also Gurhan et al. [49] concluded that a static magnetic field with different intensities affects many biological parameters such as cell growth, membrane potential, mitochondrial calcium concentration and oxidative stress in HT-1080 fibrosarcoma cells. Hence, a both polyphenols and a SMF can act synergistically together. Furthermore, we previously demonstrated their synergistic effect in inducing apoptosis in C32 cells [28] and also that a static magnetic field may be use in the design of new therapeutic strategies for diseases in the pathogenesis of which are played by disturbance of the signaling cascades that are associated with the activity of TGFβ [50]. Their common effect on cells is rather not due to the influence of a SMF on the structure of molecules [51], but it results from their similar mechanisms of action. Moreover, it was found that a high magnetic field influences the regulation of cell signaling pathways by impact on the diffusion of di- and paramagnetic molecules in cytoplasm of cells [52].

In our study, we showed that a SMF together with the tested acids may support the melanoma treatment, and thus reduces the development of cancer, through modification of *TGFβ* expression. We have shown that in cells that have been exposed to both factors: phenolic acids and a SMF, expression of TGFβ-1 and − 2 was decreased, which is best illustrated by changes in the protein level after 24 h. In turn, mRNA level of these molecules and their receptors was normalized at the end of experiment. Therefore, no statistical differences at mRNA level were noted [53]. A similar trend in the direction and dynamics of changes in mRNA and protein expression was observed by Cheng et al. [54]. They indicated that mRNAs and proteins are modulated with different temporal patterns and they observed the restoration of mRNA levels close to pre-treatment levels. On the other hand, the direction of changes in the level of protein expression was unidirectional [54]. The authors of the review by Liu et al. [55] reached similar conclusions, who considered many possibilities of the observed situation.

It should also be emphasized that in research on gene expression using various techniques, including RT-qPCR, the number of biological replicates is very important. However, the majority of gene expression studies is limited to three replicates which may affect the statistical power [56]. On the other hand, Udvardi et al. [57] and Rieu and Powers [58] found that experiments based on RT-qPCR technique should include at least three biological replicate to facilitate statistical analysis of data. Another important factor affecting gene expression profile and cell metabolism is cell confluency. Hence, the time of experiment and time of cell treatment with tested factors is very essential. Often in in vitro experiments the treatment time is 24 h.

Taken together, our study results demonstrate the potential of polyphenols and a moderate-strength static magnetic field to support cancer therapy by altering *TGFβ* expression, which is a very promising topic for the diagnosis and treatment of melanoma. It should be noted that although our studies show the potential use of phenolic acids and a SMF in melanoma, they have limitations. The use of a positive control would increase the value of the results, which should be taken into account when planning further experiments. Moreover, further research should be extended to the effects of phenolic acids and a SMF with various magnetic induction on other signaling molecules and also other cancer cell lines.

## Abbreviations

AM: acral melanoma
C32 cell line: amelanotic melanoma
CA: caffeic acid
CGA: chlorogenic acid
CM: cutaneous melanoma
CMF: combined magnetic field
COX-2: cyclooxygenase
Jak/STAT: Janus kinase/signal transducer and activator of transcription
MNP: magnetic nanoparticles
NF-κB: nuclear factor kappa B
ROS: reactive oxygen species
SMF: static magnetic field
RT-qPCR: reverse transcription-quantitative polymerase chain reaction
TGFβ: transforming growth factor β
